# Decrease in tick bite consultations and stabilization of early Lyme borreliosis in the Netherlands in 2014 after 15 years of continuous increase

**DOI:** 10.1186/s12889-016-3105-y

**Published:** 2016-05-23

**Authors:** Agnetha Hofhuis, Sita Bennema, Margriet Harms, Arnold J. H. van Vliet, Willem Takken, Cees C. van den Wijngaard, Wilfrid van Pelt

**Affiliations:** Epidemiology and surveillance unit, Centre for Infectious Disease Control Netherlands, National Institute for Public Health and the Environment (RIVM), Bilthoven, The Netherlands; Environmental Systems Analysis Group, Wageningen University, Wageningen, The Netherlands; Laboratory of Entomology, Wageningen University, Wageningen, The Netherlands

**Keywords:** Tick bites, Erythema migrans, Early Lyme borreliosis, Incidence

## Abstract

**Background:**

Nationwide surveys have shown a threefold increase in general practitioner (GP) consultations for tick bites and early Lyme borreliosis from 1994 to 2009 in the Netherlands. We now report an update on 2014, with identical methods as for the preceding GP surveys.

**Methods:**

To all GPs in the Netherlands, a postal questionnaire was sent inquiring about the number of consultations for tick bites and erythema migrans diagnoses (most common manifestation of early Lyme borreliosis) in 2014, and the size of their practice populations.

**Results:**

Contrasting to the previously rising incidence of consultations for tick bites between 1994 and 2009, the incidence decreased in 2014 to 488 consultations for tick bites per 100,000 inhabitants, i.e., 82,000 patients nationwide. This survey revealed a first sign of stabilization of the previously rising trend in GP diagnosed erythema migrans, with 140 diagnoses per 100,000 inhabitants of the Netherlands. This equals about 23,500 annual diagnoses of erythema migrans nationwide in 2014.

**Conclusions:**

In contrast to the constantly rising incidence of GP consultations for tick bites and erythema migrans diagnoses in the Netherlands between 1994 and 2009, the current survey of 2014 showed a first sign of stabilization of erythema migrans diagnoses and a decreased incidence for tick bite consultations.

## Background

Lyme borreliosis is an infectious disease caused by the bacterium *Borrelia (B.) burgdorferi* sensu lato and is transmitted through the bite of infected ticks, mainly *Ixodes ricinus* ticks in Europe. Early localised infection is typically manifested as erythema migrans, a slowly expanding red or bluish-red skin lesion that is clinically diagnosed and generally treated successfully with oral antibiotics [1]. If the diameter of the lesion is less than five centimetre, the clinical diagnosis of erythema migrans requires a history of tick-bite, a delay in appearance after the tick bite of at least two days, and an expanding rash at the site of the tick-bite. If left untreated, disseminated Lyme borreliosis can develop (e.g., Lyme neuroborreliosis or Lyme arthritis) [1], causing a considerably larger disease burden [2]. Of the various clinical presentations of Lyme borreliosis, erythema migrans is the most common, accounting for about 60 to 95 % of diagnosed Lyme borreliosis in Europe [3–8]. In the Netherlands, the relative proportions for Lyme borreliosis diagnoses in 2010 were 91 % erythema migrans, 5.3 % disseminated Lyme borreliosis (mainly Lyme arthritis 2.1 %, Lyme neuroborreliosis 1.8 %, and acrodermatitis chronica atrophicans 0.8 %), and 3.8 % persisting symptoms that were attributed to Lyme borreliosis [8]. Periodic nationwide cross-sectional retrospective studies among general practitioners (GPs) have shown a continuing and strong increase in GP consultations for tick bites and early Lyme borreliosis (measured as erythema migrans diagnoses) between 1994 and 2009 in the Netherlands [9–12]. A threefold increase was observed for the incidence of tick bite consultations from 191 per 100,000 in 1994 to 564 per 100,000 inhabitants in 2009, and concurrently the incidence of erythema migrans diagnoses increased from 39 to 134 per 100,000 inhabitants [9, 12]. Increases of Lyme borreliosis during the past decades have also been reported from other European countries [13] and from the North American continent [14, 15]. Possible explanations for these increases in the Netherlands are changes in the landscape that may have provided favourable conditions for ticks, bringing their habitat closer to humans, and increased total numbers of *Ixodes ricinus* ticks infected with *B. burgdorferi* sensu lato in the Netherlands, possibly due to increased abundance of wildlife hosts [16]. We currently report an update on the incidence of GP consultations for tick bites and erythema migrans diagnoses in 2014, assessed with identical methods as the preceding GP surveys for 1994, 2001, 2005 and 2009 [9, 12].

## Methods

In December 2014, all 10,250 GPs in our country were asked to complete a brief postal questionnaire, and in January 2015 reminders were sent to non-responding GPs. With pre-coded response categories, the questionnaire inquired about the number of consultations for tick bites and diagnoses of erythema migrans in 2014, and the size of their practice population. Values were assigned to the pre-coded response categories, based on the best fit of an assumed underlying negative binomial distribution. As every person in the Netherlands is registered with only one GP, the GP practice populations were used to calculate incidence rates per 100,000 and total numbers among the 16.8 million inhabitants of the Netherlands in 2014. Bootstrap analysis (10,000 resamplings of the GP reports) was used to calculate 95 % confidence intervals (95%CI) for the incidence rates. Two point estimates with just non-overlapping 95%CI are a conservative estimate of being different at the 0.05 level of statistical significance. More extensive method descriptions can be found in publications on preceding GP surveys [9, 12]. Ethical approval was not required for this study, because the Medical Research Involving Human Subjects Act does not apply to this type of study, collecting physician-reported counts of patients diagnosed with Lyme borreliosis and counts of tick bite consultations. To examine a possible bias of our point estimates through declining GP response over the survey years, we compared incidence estimates for tick bite consultations and erythema migrans diagnoses, stratified for the GP practices that responded to 1–2, 3–4, or all 5 of our GP surveys. We also compared predicted incidence estimates for tick bite consultations and erythema migrans for a population coverage of 60, 75 and 90 %. We did so for each survey year independently, with a simple regression model, using for each municipality the number of reported erythema migrans diagnoses (or tick bite consultations), the patient population covered by the reporting practices as offset and the response being the fraction of the municipality population covered by the reporting practices.

To explore a possible relation between the dynamics in *Ixodes ricinus* populations in the Netherlands and the dynamics of GP consultations for tick bites and erythema migrans diagnoses, we included an additional analysis on changes in tick collections from the field between 2009 and 2014. In 2006, Wageningen University started a long-term tick monitoring program in the context of the Dutch phenological network “Nature’s Calendar”. Ticks were collected by groups of trained volunteers at 12 fixed forest plots of 200 square meters each distributed across the country [17]. From 2006 to the present, ticks have been collected in the first seven days of each month, year round. The *T*-test was applied for comparison of the average annual numbers of *Ixodes ricinus* (larvae, nymphs and adults, averages calculated separately for each life stage) per field site per month. All ticks were recounted and identified to species and life stage by specialists at Wageningen University [17].

## Results

**Fig. 1:**
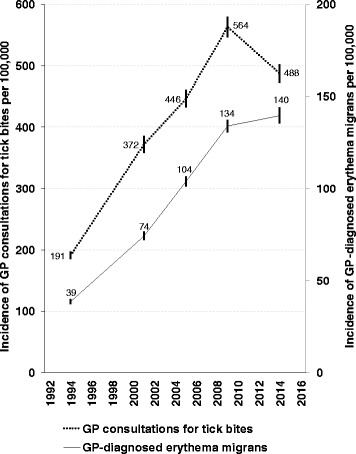
Incidence of general practitioner (GP) consultations for tick bites (left Y-axis) and erythema migrans diagnoses (right Y-axis) per 100,000 inhabitants of the Netherlands between 1994 and 2014. Point estimates with just non-overlapping 95 % confidence intervals (represented by the vertical bars) are a conservative estimate of being different at the 0.05 level of statistical significance

**Fig. 2:**
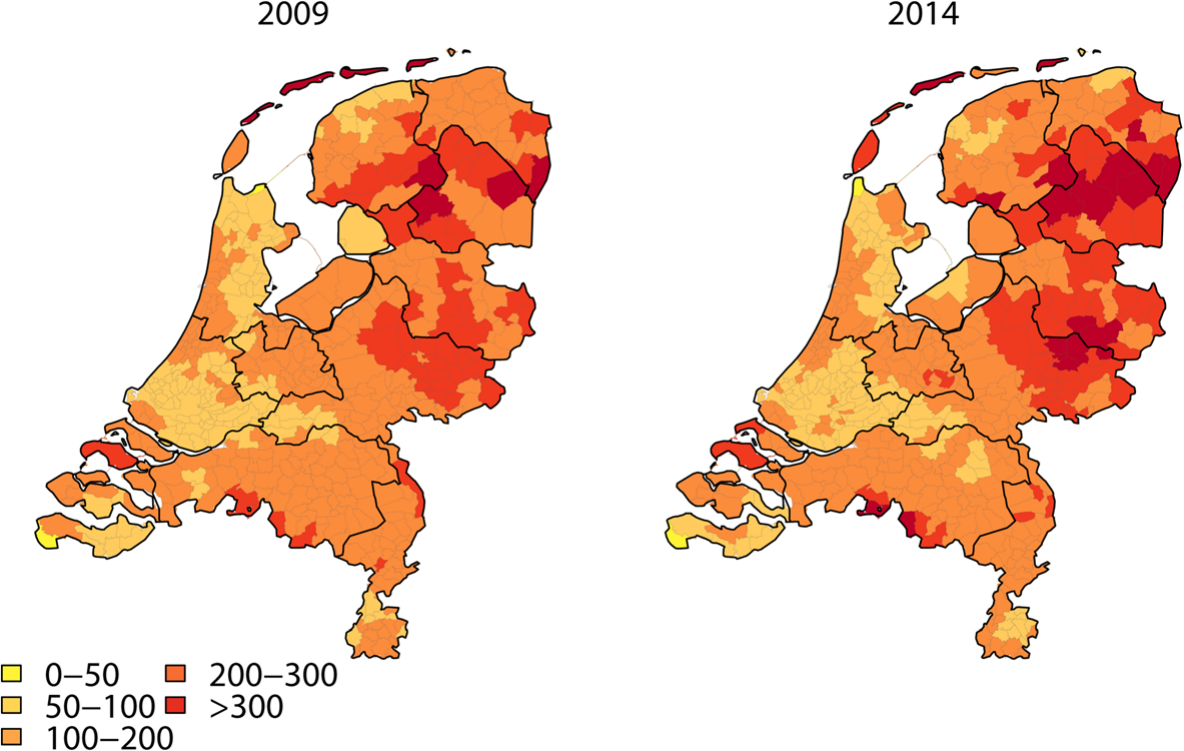
The geographical distribution of the number of GP-diagnosed erythema migrans per 100,000 inhabitants of the Netherlands in 2009 [12] and 2014

Figure 1 shows the incidence rates for GP consultations for tick bites and erythema migrans diagnoses in 2014, together with incidence rates from the preceding surveys. The four identical GP surveys of 1994 to 2009 show a continuously rising trend in consultations for tick bites and erythema migrans diagnoses. In 2014 the incidence of GP consultations for tick bites was 488 (95 % CI 473–503) per 100,000 inhabitants of the Netherlands, showing a decreased incidence for the first time since the start of these identical GP surveys in 1994. In 2014 we observed a further rise of the incidence of erythema migrans diagnoses to 140 (95%CI 135–144) per 100,000 inhabitants of the Netherlands, although the slope of the increase has decreased between 2009 and 2014. As the 95 % CI of the incidence rates in 2009 and 2014 overlap, these point estimates cannot be considered to be significantly different. All GPs nationwide saw approximately 82,000 patients with tick bites and 23,500 patients with erythema migrans in 2014. Figure 2 shows the geographical distribution of the incidence of erythema migrans diagnoses in 2014 compared to the preceding GP survey of 2009 [12]. The largest increase of erythema migrans incidence was observed in the Northeast of the Netherlands, and several locations along the West coast.

4203 GPs responded to our postal questionnaire comprising a practice population of 10.5 million persons, which covers 62 % of the 16.8 million inhabitants of the Netherlands in 2014. Table 1 shows the population coverage and incidence rates for GP-diagnosed erythema migrans in the identical surveys from 1994 to 2014. Compared to the mean incidence estimates for the whole of the Netherlands, incidence rates for erythema migrans diagnoses (see Table 1) and consultations for tick bites (not shown) did not differ substantially, nor with statistical significance, among the GP practices that responded to 1–2, 3–4, or all 5 GP surveys. However, among the GP practices that responded to all 5 GP surveys, the increasing trend in erythema migrans incidence is stronger between 2009 (132 per 100,000) and 2014 (151 per 100,000), with non-overlapping 95 % CI. The predicted incidence rates with population coverage set at 60 %, 75 % or 90 % also did not differ substantially, or with statistical significance, compared to the mean incidence estimates for the whole of the Netherlands. The predicted incidence rates for erythema migrans with population coverage set at 60, 75 or 90 % each showed a rising incidence with non-overlapping 95 % CI between 1994 and 2009, and a stabilizing incidence of erythema migrans in 2014, as was observed with the mean incidence estimates. With lower population coverage, our model predicted slightly lower incidence rates. So the decline in population coverage over the years probably did not measurably bias our incidence estimates, if yet, only the 95 % CI around the estimate have slightly broadened (see Fig. 1).

**Table 1:** Annual incidence of erythema migrans (EM) diagnoses per 100,000 inhabitants of the Netherlands for each survey year, predicted for varying response rates, and stratified for the GP practices that responded to 1 to 2, 3 to 4, or all 5 of our GP surveys

Survey year	1994	2001	2005	2009	2014
% Population coverage	88 %	68 %	71 %	65 %	62 %
Mean incidence of EM	38.6 (37.2–40.0)	74.3 (72.0–76.6)	103.8 (101.0–106.6)	133.9 (130.5–137.5)	139.6 (135.3–144.1)
Incidence of EM (and 95 % CI) per number of GP practices responding to 1 to 5 surveys:					
1 to 2 surveys	39.0 (36.7–41.4)	72.3 (66.9–78.1)	112.9 (102.6–123.8)	131.7 (124.8–138.8)	133.7 (127.5–140.2)
3 to 4 surveys	38.3 (35.8–40.9)	77.9 (74.6–81.2)	102.0 (89.3–105.9)	136.6 (131.0–142.3)	137.7 (130.2–145.8)
5 surveys	38.5 (36.2–40.8)	73.0 (69.2–77.2)	104.0 (99.4–109.0)	132.3 (126.3–138.4)	150.6 (141.5–160.1)
Incidence of EM (and 95 % CI) among municipalities with population coverage:					
60 % coverage	40.2 (32.5–49.0)	73.0 (67.6–78.8)	99.6 (91.4–108.8)	132.3 (123.5–142.7)	139.8 (129.1–152.0)
75 % coverage	39.5 (35.2–44.6)	76.3 (71.2–81.8)	104.5 (98.4–111.1)	135.7 (127.6–144.7)	141.0 (131.5–151.8)
90 % coverage	38.8 (36.4–41.5)	79.5 (71.2–88.5)	109.5 (101.2–118.0)	139.0 (127.8–150.7)	142.1 (129.7–155.9)

In the long-term tick monitoring program of the Wageningen University, no statistically significant increases or decreases were observed in the average annual numbers of *Ixodes ricinus* (larvae, nymphs and adults, averages calculated separately for each life stage) collected monthly at the 12 field sites between 2009 and 2014. The average annual number of nymphs per field site per month was 231 in 2014, which was 7 % higher compared to 217 in 2009 (difference not statistically significant). The average annual number of adult ticks per field site per month was 31 in 2014, which was 11 % higher compared to 28 in 2009 (difference not statistically significant).

## Discussion & conclusion

In contrast to the constantly rising incidence of GP consultations for tick bites and erythema migrans diagnoses between 1994 and 2009, we have currently observed a first sign of stabilization of erythema migrans diagnoses and a decreased incidence for tick bite consultations in 2014. Possibly, this decrease may have started earlier after 2009, as incidence rates for tick bite consultations and erythema migrans diagnoses similar to the incidence rates in 2014 have already been reported for 2010 [8]. The 2010 incidence rates for tick bite consultations and erythema migrans diagnoses were 495 (95 % CI 478–512) and 132 (95 % CI 127–136) per 100,000 respectively, as obtained in a nationwide cross-sectional retrospective survey among physicians, using an extended questionnaire on all Lyme borreliosis manifestations, with comparable methods to the current GP survey [8]. Based on relative proportions for Lyme borreliosis diagnoses in the Netherlands [8], the currently observed 23,500 erythema migrans diagnoses likely indicate an additional 1400 diagnoses with disseminated Lyme borreliosis, plus 1000 patients with Lyme-related persisting symptoms, resulting in a total of 25,800 cases with diagnosed Lyme borreliosis in 2014. Based on a previous study in which we compared the GP reports from 2005 to tick bites reported by the general population of the Netherlands, we observe one tick bite consultation for approximately every thirteen tick bites among the general population of the Netherlands (ratio 7.8 %) and we observe one GP-diagnosed erythema migrans for approximately every sixty tick bites among the general population of the Netherlands (1.8 %) [12]. This ratio for tick bites among the general population versus erythema migrans diagnoses is comparable to the risk estimates for developing erythema migrans after tick bites as observed in prospective studies [18–20].

During the past decades, increases of Lyme borreliosis have been reported from European countries such as the United Kingdom, the Czech Republic and Hungary [21–23]. In Germany, the direct Eastern neighbour of the Netherland, the incidence of Lyme borreliosis increased between 2001 and 2006, and stabilized between 2009 and 2012 [7, 24]. South to the Netherlands, in Belgium and France incidence rates for Lyme borreliosis remained stable between 2003 and 2012 [3, 25, 26].

The greatest increase in erythema migrans incidence was observed in the Northeast of the Netherlands and several locations along the West coast, between 2009 and 2014 (Fig. 2) as was seen between 1994 and 2009 [12]. This geographical distribution of high incidence regions for erythema migrans diagnoses is strongly correlated with tick presence as predicted through environmental risk mapping [27]. Geographically focal increases in erythema migrans incidence could be due to increases in the risk of infection with *B. burgdorferi* sensu lato after tick bites, or specifically *B. afzelii* which is mostly associated with skin manifestations such as erythema migrans and acrodermatitis chronica atrophicans [28]. However, there is no evidence for rising prevalence of *B. burgdorferi* sensu lato in ticks, based on combined analysis of two large field studies [16]: one longitudinal series of tick collections between 2000 and 2009 in a coastal dune area [29], and the long-term tick monitoring program of Wageningen University geographically spread throughout the Netherlands between 2006 and 2009 [17].

For the first time since the start of our identical GP surveys in 1994, we have currently observed a decreased incidence for tick bite consultations. Our analyses of the average annual numbers of ticks per field site per month from the tick monitoring program of Wageningen University did not indicate a significant change in *Ixodes ricinus* abundance in the Netherlands between 2009 and 2014. While erythema migrans is a clear sign for a patient to seek medical care, most tick bites do not require a GP’s attention. Whether people decide to consult a physician for a tick bite, rather than remove the tick themselves, may be influenced by having tick bites frequently, public health education and media attention.

Since the outcomes of the first GP survey on 1994 were reported, the National Institute of Public Health and the Environment of the Netherlands (RIVM), and Wageningen University, together with other stakeholders, have prompted extensive national media attention on Lyme borreliosis each year around springtime, with a national awareness week on tick bites (www.weekvandeteek.nl) to mark the onset of the tick bite season. The RIVM distributed annually updated campaign materials for public health education of the general population on the prevention of tick bites and Lyme borreliosis, and published a national guideline for professionals on the prevention, diagnosis, and treatment of Lyme borreliosis [30]. The RIVM redesigned the campaign materials for public health education of the general population in 2011, focussed on skin checks and removing ticks - which the public perceived most feasible [31], whereas previously the campaign materials presented all possible evidence based preventive measures. In 2012 the RIVM started approaching the public through social media, and with an educational online video [32], and school-aged children were targeted with an online serious game, teaching them about ticks and Lyme borreliosis in a playful way [33]. In 2014 a mobile phone app on ticks and Lyme borreliosis was launched. The effectivity of the educational online video, online game and the mobile phone app are being evaluated, to be published by Beaujean et al. Awaiting the study outcomes on effectivity of public health education, we tentatively propose that the decrease in GP consultations for tick bites may reflect the impact of repeated and redesigned efforts of public health education about the relevance of body checking and prompt tick removal and when to visit a physician. Further monitoring and analysis of the dynamics between humans and *Ixodes ricinus* (infected with *B. burgdorferi* sensu lato), is required to identify reasons for the currently observed change in trend after 15 years of continuous increase of GP consultations for tick bites and erythema migrans diagnoses in the Netherlands.

## Abbreviations

95 % CI: 95 % confidence intervals
*B. burgdorferi*: *B. afzelii, Borrelia burgdorferi* sensu lato, *Borrelia afzelii*
EM: erythema migrans
GP(s): general practitioner(s)
RIVM: National Institute for Public Health and the Environment, of the Netherlands
